# Structure and Biomechanics of the Endothelial Transcellular Circumferential Invasion Array in Tumor Invasion

**DOI:** 10.1371/journal.pone.0089758

**Published:** 2014-02-24

**Authors:** Constadina Arvanitis, Satya Khuon, Rachel Spann, Karen M. Ridge, Teng-Leong Chew

**Affiliations:** 1 Department of Cell and Molecular Biology, Northwestern University Feinberg School of Medicine, Chicago, Illinois, United States of America; 2 Center for Advanced Microscopy, Northwestern University Feinberg School of Medicine, Chicago, Illinois, United States of America; 3 Division of Pulmonary and Critical Care Medicine, Northwestern University Feinberg School of Medicine, Chicago, Illinois, United States of America; Dalhousie University, Canada

## Abstract

Cancer cells breach the endothelium not only through cell-cell junctions but also via individual endothelial cells (ECs), or transcellular invasion. The underlying EC forms a circular structure around the transcellular invasion pore that is dependent on myosin light chain kinase (MLCK) and myosin II regulatory light chain (RLC) phosphorylation. Here we offer mechanistic insights into transcellular invasive array formation amid persistent tensile force from activated EC myosin. Fluorescence recovery after photobleaching (FRAP) experiments, sarcomeric distance measurements using super-resolution microscopy and electron microscopy provide details about the nature of the myosin II invasion array. To probe the relationship between biomechanical forces and the tension required to maintain the curvature of contractile filaments, we targeted individual actin-myosin fibers at the invasion site for photoablation. We showed that adjacent filaments rapidly replace the ablat11ed structures. We propose that the transcellular circumferential invasion array (TCIA) provides the necessary constraint within the EC to blunt the radial compression from the invading cancer cell.

## Introduction

The entry of cancer cells into the bloodstream is a critical point in the metastatic cascade [1] and usually leads to poor prognosis [2], [3]. The molecular events governing transendothelial migration are poorly characterized, yet they present great opportunities for potential therapeutic intervention. Recently ECs have been implicated in actively facilitating tumor transmigration. Cancer cells can alter the biomechanical properties of ECs, which may in turn modulate the invasive potential of cancer cells [4], [5]. In addition, cancer cells also directly regulate the biochemical signaling within the underlying endothelium [6].

More importantly, metastatic breast cancer cells are capable of breaching the endothelium through two distinct mechanisms: (i) paracellular invasion, wherein cancer cells disrupt the EC border; (ii) transcellular invasion in which the cancer cells penetrate individual ECs without causing cell death [7], [8]. Interestingly, transcellular migration is not restricted to tumor invasion but also occurs frequently in leukocyte diapedesis. Both leukocyte and cancer transcellular migration share parallel features, implicating the presence of a potentially conserved mechanism within the EC. Several groups have shown that the EC apical surface exhibits significant membrane dynamics upon initial interaction with transmigrating cells [8]–[11]. In addition, a multitude of membrane associated components also mediate membrane dynamics during transcellular invasion [12], [13]. As transcellular migration proceeds to the active phase, the EC cytoskeleton begins to reorganize and form transient structures that encapsulate the intrusive cell [8], [14]. These events provide clues about the signaling crosstalk between the transmigrating cell and the EC that converge on cytoskeletal and membranous networks.

Several groups have proposed that cancer cells are capable of mimicking leukocytes when undergoing diapedesis [15]. However, the onset of transcellular tumor invasion poses a significant survival challenge for the underlying ECs compared to leukocyte migration. A tumor cell is several-fold larger than an average leukocyte [16]. How ECs compensate for the trauma of tumor transcellular migration is an important yet poorly understood mechanism. Numerous daunting technical obstacles of studying tumor transendothelial invasion have hampered attempts to elucidate this mechanism. These include the unpredictable loci of occurrence, low transmigrating frequency (∼10% in our *in vitro* condition), ∼10-fold time lag to the initiation of invasion compared to leukocyte migration, and the demand of high spatio-temporal resolution imaging needed to decipher the underlying mechanisms. As a result, to the best of our knowledge there has only been one other study on the EC cytoskeletal rearrangements that occur during cancer invasion, specifically via the transcellular route [8].

The most damaging intracellular trauma incurred during transcellular invasion is the shear and compressive forces imparted by the transmigrating cells, especially considering the larger size of cancer cells compared to leukocytes. To prevent itself from bursting from within, ECs must devise a counter-mechanism that is viscoelastic yet flexible to withstand transmigration. We have reported that interaction with cancer cells induces localized MLCK activity and contractile function in the underlying EC [8]. However, several aspects of the EC response to transcellular invasion remain poorly understood. First, it is unclear what underlying molecular mechanism governs the formation of the myosin-rich transcellular circumferential invasion array (TCIA) that encapsulates the invasion pore: Is it a result of *de novo* myosin assembly or *in situ* reorganization? Second, the optical resolution limit had previously prevented our ability to elucidate the architecture of myosin within the TCIA. It is unclear if myosin is arranged in a manner comparable to regular stress fibers. Third, it remains to be elucidated if the invasion event would modulate the myosin turnover rate within the TCIA as compared to stress fibers, a potentially informative indicator for functional and organizational distinction. Fourth, the important structure and function relationship of the TCIA remains unexplored to date. It is interesting to consider how an active contractile system maintains its curvature, as well as how this structure contributes to its function in facilitating transcellular invasion, and vice versa. What would the consequence be if the integrity of the TCIA were locally perturbed?

In this study we address these questions by investigating the (i) the mechanism of TCIA formation, (ii) molecular nature of myosin within the array and (iii) the ability of the TCIA to counteract the compressive forces of the invading cancer cell. Our data reveal that the EC actively reorganizes its stress fibers to circumscribe the invasion pore with concomitant increase in myosin density and stability. Some individual stress fibers also undergo stress-induced fracture and recoil from the invasion site resulting in larger invasion pore. To investigate if the TCIA is maintained by the balance of tumor cell compressive force and EC contractile force, we selectively photoablate individual EC stress fibers immediately flanking the invasion pore and show that the adjacent stress fiber rapidly assumes the position of the displaced filament. Our results implicate the EC cytoskeleton as an important self-protective mechanism and highlight the underlying biomechanics as a key determinant of transendothelial migration.

## Results

### Invasion pore development precedes the formation of TCIA

ECs are capable of supporting both paracellular and transcellular invasion (Figure S1) [17]. During paracellular invasion there is clear disruption of VE-cadherin at the border and displaced cortical actin bundles around the invasion site. In contrast, during transcellular invasion, the border remains intact and the EC myosin is assembled into a ring-like array around the circumference of the invasion pore that encapsulates the invading cancer cell (Figure S1D). We define this structure as the transcellular circumferential invasion array (TCIA).

**Figure 1:**
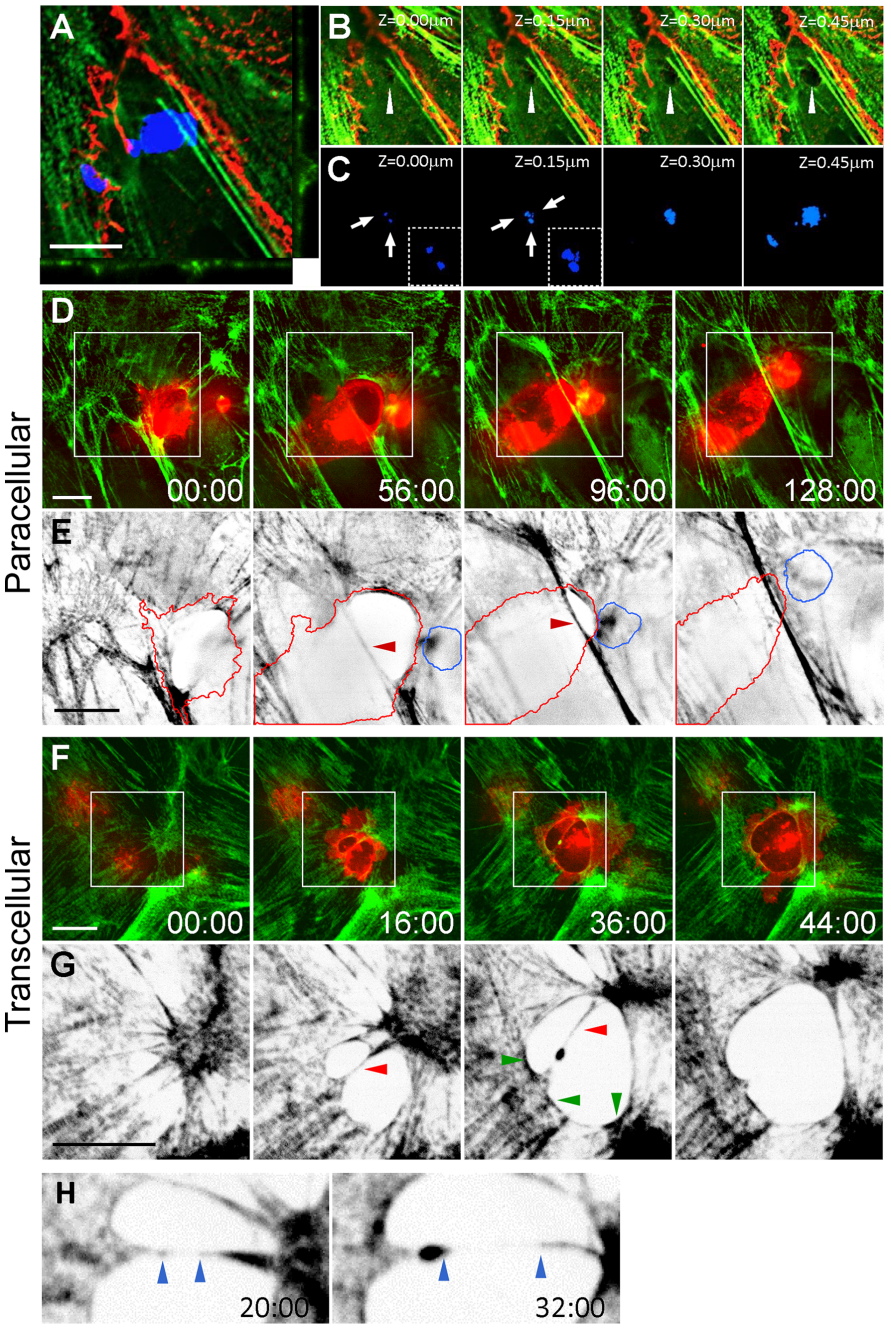
Confocal micrographs of transcellular and paracellular invasion. (A) Orthogonal view of an early transcellular invasion of MDA-MB231 cell (blue) invading an intact HUVEC monolayer expressing GFP-RLC (green) and stained for VE-Cad (red). (B) Z-sections of invasion pore (white arrowhead). (C) Corresponding Z-sections of cancer cells (arrows) penetrating the monolayer. Inset shows zoomed cancer cell invasive protrusions. Spinning disk confocal images of paracellular (D and E) and transcellular (F and G) invasion. White boxes show zoomed area in panels E and G. (E and G) Intensity-inverted images of GFP-RLC monolayer for ease of visualization. (D) Red lines  =  invading cancer cell, blue  =  uninvaded; red arrowhead highlights stress fiber. (G) Intensity-inverted images of transcellular invasion. Red arrowhead indicates bisecting stress fiber; green arrowheads highlight nascent myosin recruitment. (H) Zoomed area of G showing stretched sarcomeric spacing. Blue arrowheads indicate edges of myosin-denuded bisecting stress fiber. Time is mm:ss. Scale bar, 10 μm.

The mechanism triggering TCIA formation is poorly understood. We hypothesize that the TCIA forms as a reaction to forces generated by the invading cancer cell. If this hypothesis holds true, we should be able to detect an early tumor penetration event in which the EC cytoskeleton does not have sufficient time or appropriate signals to undergo reactionary reorganization. Indeed, we have observed early invasion events where there is lack of myosin-enriched arrays. Figure 1A shows a cancer cell (blue) at the very early stage of invasion, wherein the cancer cell probes the dorsal surface the underlying EC. Confocal micrographs show optical sections of several invasive protrusions penetrating the EC (Figures 1B and C). Closer examination of the EC cytoplasm reveals a pore devoid of a myosin ring (arrowhead) penetrated by invasive protrusions (arrows). The absence of myosin in these early invasion pores within the EC raises interesting questions about the timing, mechanism of formation, and the molecular architecture of the invasion array.

### Dynamic restructuring of the endothelial actin-myosin stress fiber during transcellular invasion

To gain mechanistic insight into the formation of the TCIA, we performed spinning disk confocal microscopy. Due to toxicity associated with overexpressing and imaging multiple fluorescently tagged proteins in HUVECs, we were unable to simultaneously image the EC borders with fluorescent VE-Cadherin (VE-Cad) for prolonged periods. Transcellular invasion is thus detected by differential interference contrast microscopy through which the EC border can be visualized (not shown). Figure 1D–G show time-lapse confocal micrographs of MDA-MB231 breast cancer cells undergoing invasion through HUVECs expressing GFP-RLC.

Figure 1D and E and Movie S1 show a cancer cell undergoing paracellular invasion. The underlying EC dynamically reorganized its cytoskeleton to accommodate the transmigration. One of the prominent actin bundles (red arrowheads) was displaced by the cancer cell to a different focal plane but remained relatively linear. This actin bundle eventually returned to the initial focal plane as the cancer cell crawled past the invasion site. Upon completion of paracellular transmigration, the cancer cell moved underneath the HUVEC layer and migrated away. The displacement of cortical actin during paracellular migration is characteristic of this process, which can be observed in over 85% of paracellular invasion events.

During transcellular diapedesis, transmigratory cells often probe the EC dorsal surface prior to migration [18], [19]. We speculate that the cancer cell employs a similar strategy to probe for weaker points in the EC cytoplasm for penetration. This is exemplified in Figure 1A–C as well as the transient formation of two invasion pores transected by a single stress fiber (red arrowheads) in Figure 1F and G and Movie S2. As the invasion progressed, the invading cell caused several significant restructuring events in the EC actomyosin system: First, the transecting stress fiber exhibited stretched sarcomeric spacing (Figure 1H, blue arrowheads) with a considerable length of the actin filament being denuded of myosin, until the fiber eventually broke and recoiled (red arrowhead) to form a much larger pore. Second, EC myosin began to circumscribe the invasion site (green arrowheads) indicating the onset of TCIA formation. To the best of our knowledge, this is the first report to describe the dynamic events during transcellular invasion that subsequently give rise to a TCIA. It is important to note that because there is currently no marker that defines the onset of invasion, the indicated time points are assigned according to our experimental imaging time. These durations are consistent with previous findings [20].

The recruitment of EC myosin to circumscribe the invasion pore and the subsequent formation of the TCIA implicate a reactionary remodeling process of EC stress fibers to cope with the intracellular microwound. Our data suggest that the local EC myosin is put under a significant level of stress during transcellular invasion and that there is locally potentiated myosin contraction. This is consistent with our previous results showing increased EC myosin II phosphorylation [8], implicating an increase in myosin filament assembly and its interaction with actin filament [21], [22]. Together, these data suggest that transcellular invasion will likely affect myosin stability within the TCIA.

### Myosin turnover within the TCIA is significantly different from normal stress fibers

**Figure 2:**
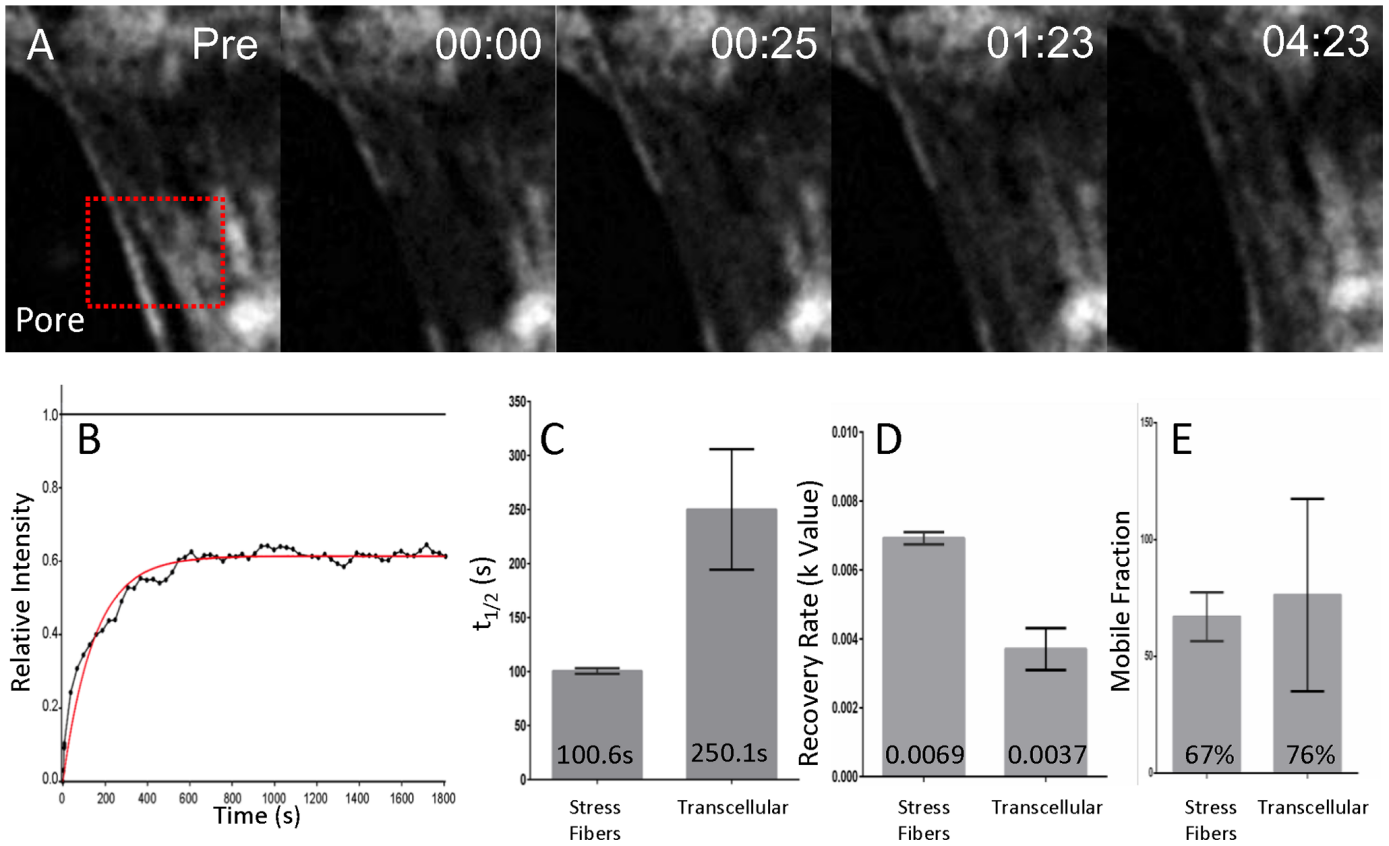
Myosin exchange with the circumferential invasion array. (A) Representative timelapse images of a FRAP zone (red box) in a transcellular pore. Time  =  mm:ss. (B) A representative fluorescence recovery curve with relative intensity normalized to pre-bleached level. (C–E) Comparison of t_1/2_, k value, and mobile fraction between stress fibers and transcellular invasion arrays. n = 8 for each bar. Mean values are displayed on graph.

To determine if myosin exhibits different kinetics within the TCIA compared to stress fibers in a non-invaded cell, we performed fluorescence recovery after photobleaching (FRAP) experiments to compare myosin turnover rates. Figure 2A shows the fluorescence recovery of the bleached zone of a representative invasion array. We quantified the t_1/2_, mobile fraction and recovery rates of myosin within stress fibers and the TCIA. Figure 2C and D show that myosin in the invasion array exhibits markedly slower turnover kinetics in t_1/2_ (*p* = 0.018) and k value (*p = *0.0009). We postulate that the decreased myosin II molecular exchange is the result of reduced myosin off-rate from the stress fibers within the TCIA. Our observation of varied t_1/2_ of myosin recovery rates is consistent with previous results showing a three fold decrease in myosin turnover rates within peripheral actin when compared to stress fibers [23], indicative of increased myosin bundling within the TCIA, or that the EC myosin may be more compressed due to potentiated contraction. Interestingly, myosin networks in both stress fibers and the invasion array showed comparable level of mobile fraction (Figure 2E), even though the invasion array recorded significantly larger standard deviation. This large variation in deviation was likely due to increased inherent difficulty in measurement as the TCIA continued to change shape and translocate with the invading cancer cells. The indistinguishable myosin II mobile fractions within the TCIA and the stress fibers show that a comparable percentage of myosin is available to participate in diffusional exchange, indicative of potentially similar molecular properties. Therefore the significant decrease in myosin turnover rate within the TCIA is not a result of myosin availability and needs to be further examined.

### The TCIA consists of densely packed, contracted stress fibers and parallel bundled actin filaments

**Figure 3:**
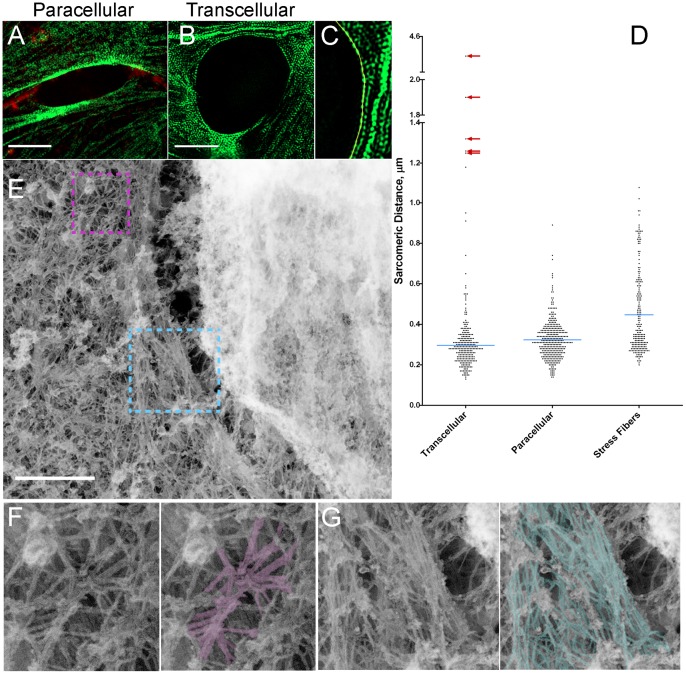
Ultrastructural studies of invasion pore. (A and B) SIM images of GFP-RLC (green) and VE-Cad (red) in HUVEC. (C) Example of intensity trace for measuring sarcomeric spacing. (D) Scatter plot of sarcomeric distances (n = 264 for transcellular, 368 for paracellular, and 218 for stress fibers). Blue lines, mean distribution. Red arrows show outliers from myosin-denuded areas of invasion arrays which were not included in the analysis. (E) Platinum replica electron microscopy of transcellular invasion. (F and G) Zoomed areas of purple and cyan boxes. Scale bars, 5 μm (A and B) and 500 nm (E).

The distinct myosin turnover rates motivated us to examine the nature of TCIA myosin arrangement with enhanced resolution. We previously observed contraction of myosin in the vicinity of the invasion pore, but were unable to resolve the high density of myosin structure within the TCIA itself beyond the light diffraction limit using conventional confocal microscopy. Here we turned to super-resolution structured illumination microscopy (SIM) to overcome this hurdle and compared myosin ultrastructure within the TCIA versus the paracellular invasion pore. As shown in Figure 3A and B, we were now able to resolve most of the dense sarcomeres within the transcellular invasion array as well as the displaced cortical actin bundles within paracellular invasion pores and regular stress fibers (Figure 3A and B). Leveraging our ability to further resolve the sarcomeres around invasion pores, we compared myosin contraction as a function of sarcomeric distances (i) along the stress fibers in non-invaded ECs, (ii) the paracellular invasion pore, and (iii) within the TCIA.

In stress fibers, myosin showed significantly more relaxed myosin periodicity with a mean sarcomeric distance of ∼447±14 nm. On the other hand, along paracellular invasion pores, EC myosin exhibited a considerable degree of contraction (mean sarcomeric distance ∼323±5.0 nm). In contrast, the sarcomeric distance within the TCIA, a measurement only now made possible with super-resolution microscopy, registered a significantly contracted state (mean sarcomeric distance ∼296±6.5 nm).

The sarcomeric distance within normal stress fibers in non-invaded ECs shows a wide distribution of values and a weak clustering around a median of 350 nm. The large range of stress fiber-related sarcomeric distance was expected because myosin contraction along stress fibers had been revealed to be non-uniform [23]. In our measurement of sarcomeric distances along stress fibers in non-invaded cells, we did not preferentially select stress fibers in specific cellular regions. Therefore our measurements will comprise of myosin potentiated to different extents. In contrast, myosin II spacing in the vicinity of tumor invasion (regardless of invasion routes) exhibited significantly tighter distribution when compared to regular stress fibers (p<0.0001), indicating that EC contractile machinery was generally activated in response to local force perturbation during tumor invasion.

The difference in sarcomeric gaps between the TCIA and the paracellular invasion pores, while statistically significant (p = 0.03), is smaller than expected. However, the mean sarcomeric gap of 296 nm within the TCIA is 25% narrower than the shortest mean distance recorded in the literature [23]. More importantly, the TCIA sarcomeric distance matches the length of individual non-muscle myosin II thick filaments, as measured using electron microscopy [24], [25]. Our data therefore suggest that the EC myosin has reached the shortest possible sarcomeric distance within the TCIA, hence maximal contraction. Interestingly, in addition to the fully contracted myosin, the distribution plot (Figure 3D) also showed numerous outliers (red arrows), in which EC myosin exhibited wider than expected myosin gaps. Some of these gaps easily span the distance of 8–12 sarcomeric distances, which we believed to correspond to areas that were denuded of myosin due to excessive stretching by the invading cancer cell. This phenomenon is exemplified previously by Figure 1H and Movie S2.

To further characterize the architecture of the TCIA, we performed platinum replica transmission electron microscopy [26], [27]. Figure 3E shows a dense arrangement of actin bundles around the cancer cell in the invasion array. Detailed tracing of the actin filaments highlighted the long and parallel nature of the fibers along the pore (Figure 3G, blue), compared to many actin filaments in the vicinity that remained radially organized (Figure 3F, purple).

### Invasion arrays are not perfectly circular, and contain gaps between the ECs and the cancer cells

The actomyosin network of stress fibers and cortical actin bundles are assembled to physiologically provide tensile strength within EC. Due to the intrinsic isometric tension [28], stress fibers are usually pulled into linear filaments with viscoelastic properties [29]. The formation of the TCIA thus raised an interesting biomechanical expectation. If the intrinsic isometric tension were the sole contributing factor, then two predictions would follow. It would dictate the existence of a circular network around the micro-wound caused by the invasion [30]. Also, there would be no space between the cancer cell and the invasion array. Surprisingly, we found no data that were consistent with either prediction.

**Figure 4:**
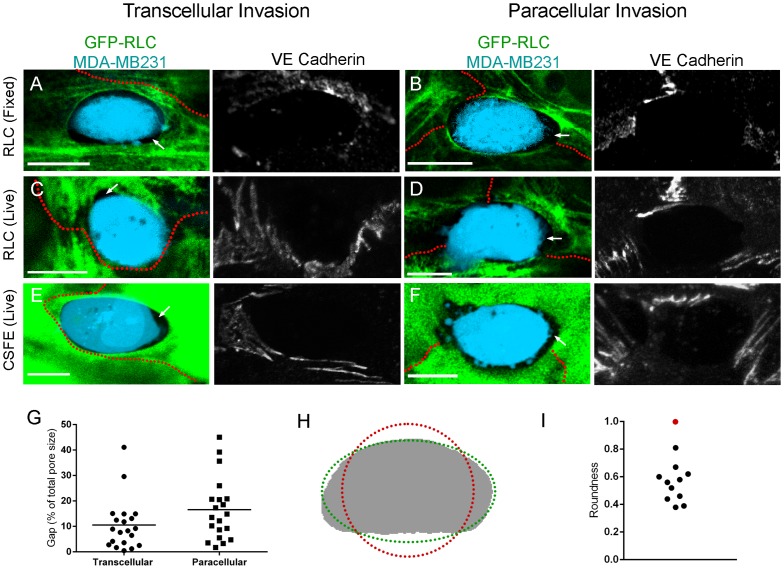
Invasion Array Shape Analysis. (A–F) Confocal micrographs of gaps between cancer cells (blue) and EC under 3 different conditions. MDA-MB231 are labeled with CellTrace Violet. Maximum intensity projections of VE-Cad delineating cell borders in gray scale. (A and B) Fixed EC and (C and D) live EC expressing GFP-RLC. (E and F) Live EC labeled with CSFE. White arrows show intercellular gaps. Red outlines indicate EC border as determined by VE-Cad stain. (G) Area of gap between EC and invading cancer cell, n = 20 per group. (H) Outline of an invasion pore (gray) fitted to a circle (red) and an ellipse (green). (I) Roundness factor from 11 transcellular arrays (black) and a perfect circle (red). Scale bar, 10 μm.

First, gaps were visible between the invasion array and the cancer cell (Figure 4A–F). Figure 3E and Figure 4A–F show that these gaps can be clearly visible at both the electron and light microscopy levels. This unexpected phenomenon was not a result of artifacts due to microscopy focused only on GFP-RLC, or specimen shrinkage from fixation. Using live cytoplasmic stains, we also observed these gaps during imaging of live invasions (Figure 4E and F). While the size of the gap varied significantly (Figure 4G), over 84% of invasions both in live and fixed cells showed gaps (n = 74).

Equally striking and reproducible was the absence of circularity in the TCIA. In fact, most arrays showed considerable variance in the local radii of curvature. Figure 4H shows a representative invasion pore to demonstrate our subsequent measurements. We obtained the shape indices of 11 transcellular invasion arrays and the roundness factors were plotted in Figure 4I. A perfect circle would have a roundness of 1 (red dot). As demonstrated, these pores defied attempts to fit a circle within them. There was a wide variation in the arc angles within the invasion pores (Figure 4A–F), thus revealing the segmental and uneven tension distribution along the actin-myosin array around the invading cells. To reconcile myosin contractile force with the irregularly shaped TCIA, we focused our investigation on regional biomechanical forces that may locally shape the invasion-driven structure.

### Biomechanical forces dictate the structure of actin-myosin network within the TCIA

**Figure 5:**
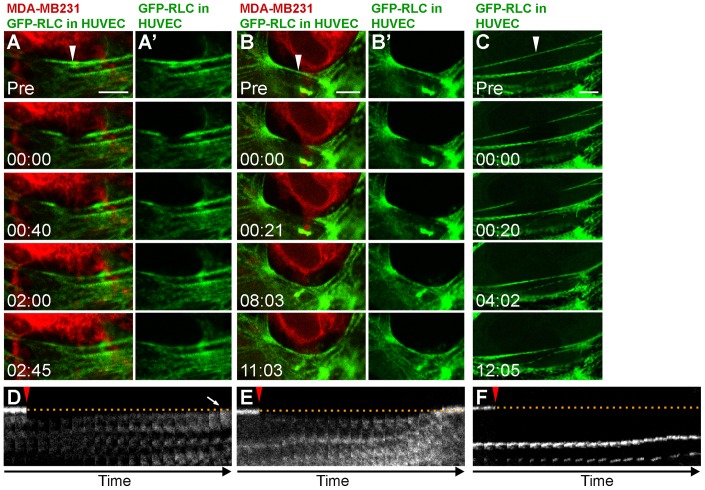
Biomechanics of invasion pore. (A and B) Laser ablation of GFP-RLC stress fibers (green) in HUVEC during MDA-MB231 (red) invasion. White arrowheads indicate ablation point. (A' and B') GFP-RLC alone is shown. (C) Ablation of a stress fiber in a resting EC. (D–F) Kymographs of ablated zones. Dotted line shows initial positions of ablated fibers. Ablation pulse is indicated by red arrowheads. Time is mm:ss. Scale bar, 5 μm.

If the invasion array is a tension-driven structure with stress exerted by the invading cancer cell, then the network cannot tolerate a local perturbation of the balanced forces and will therefore undergo rapid remodeling to compensate for the imbalance in tension. Conversely, if the TCIA was not actively counter-balancing the tension against the invading cancer cell, then a perturbation of a single stress fiber would likely have little to no effect on the TCIA structure. To test this hypothesis, we performed laser micro-photoablation. We specifically severed stress fibers at the site of cancer invasion, resulting in immediate scission of the targeted fibers (Figure 5, A and B; Movies S3–S4). The immediate viscoelastic recoil of the stress fiber away from the ablation zone indicated that this was an actual physical breakage and not photobleaching [31]. As postulated, local remodeling of endothelial stress fibers occurred rapidly following photoablation. The adjacent stress fiber within the invasion array shifted to the position of the fiber displaced by photoablation (Figure 5A'). The kymograph shows the adjacent stress fiber assuming the exact position (arrow, Figure 5D) of the severed fibers. This result suggests that the regional radius of curvature within the invasion array is determined by the contractile force of the EC actomyosin reacting to locally counteract the compressive and shear forces incurred by the invading cell. We also performed photoablation on the same spot from Figure 5A in succession upon completion of the stress fiber remodeling. As predicted, the next adjacent stress fiber repositioned to take the place of the one destroyed by photoablation (Movie S3). Likewise, we also micro-ablated a myosin fiber within a region of the invasion array that was linear and contained gaps between the cancer cell and the EC. The myosin fiber was also restored to the position matching the linear configuration of the pre-ablation fiber as shown in Figure 5B', and further confirmed by kymograph in Figure 5E, suggesting that there was a compensatory transference of tensile loads borne by the pre-ablated fibers to the adjacent fiber upon scission. In contrast, we did not observe similar repositioning of adjacent fiber when stress fibers were ablated in resting ECs (Figure 5, C and F; Movie S5).

## Discussion

Transendothelial migration of both cancer cell and leukocytes can be mechanistically divided into three distinct stages as defined by the response of the underlying endothelium. The **early phase** is marked by invasive protrusions of cancer cells or podosomes of leukocytes probing of the endothelial cell [19], and a lack of mature myosin II-enriched circular invasion pore network around the entry site, as demonstrated in this report. The **active transmigrating phase** occurs when the underlying EC begins to envelope the incoming transmigrating cell. In the case of cancer migration, this is prominently marked by a circumferential invasion array comprised of a high density of actin and myosin II surrounding the tumor invasion pore, with active myosin contraction potentiated at least in part by localized MLCK activity [8]. Whether or not endothelial actomyosin also reorganizes into ring-like structure around the site of leukocyte transcellular diapedesis remains a topic of debate [9], [11], [12]. Recently, Martinelli *et al.* [10] have presented compelling evidence detailing the **late phase** of transcellular diapedesis upon the exit of the transmigrating cell, wherein ventral lamellipodia, a newly discovered actin-rich structure, seals the pore or “micro wounds” of the endothelial cell as a form of recuperative response.

We have previously postulated that the TCIA would be a transiently assembled *de novo* structure [8]. The logic of this argument was based on the acute curvature of the array that appeared incongruent with an actively contracting, high tensile structure. Our current study therefore seeks to unravel, from the perspective of the ECs, the mechanistic events that breach the endothelial barrier during the active transmigrating phase. In this study we dissected the dynamic remodeling of the cytoskeleton within the ECs and the nature of this intracellular passageway by examining its process of formation, the molecular nature of its ultrastructure, as well as the determining factor that creates the curvature of a contractile structure with intrinsic tensile force. We propose that the TCIA is formed by local reorganization of existing EC actomyosin fibers, often as a result of stretching, displacement, and breakage by the invading cancer cells. Data presented here that support this notion include time-lapse observation, ultrastructural examination, indistinguishable myosin mobile fractions following photobleaching, and micro-photoablation. However, our current data cannot exclude the possibility of myosin recruitment through unidentified mechanisms.

Detailed sarcomeric measurement using structured illumination microscopy indicates that the EC myosin is highly contracted within the TCIA. In fact, the myosin gap we measured approached the length of a bipolar myosin thick filament [24], as measured by electron microscopy. We believe this is of biological importance as it suggests that the EC myosin within the TCIA has undergone maximal contraction. In our previous study, we showed that EC myosin II underwent significant contraction at a rate of 50% sarcomeric shortening within 5 min in the vicinity of an established TCIA [8]. At that time, we were unable to get optically close to the TCIA due to diffraction limit, restricting our ability to effectively resolve the densely packed actomyosin network at the invasion pore. Together with the significantly decreased turnover rate of TCIA myosin II, we propose that the invasion array consists of densely compacted, and highly contracted actin-myosin network. The increasing gradient of EC myosin contraction toward the TCIA allows us to postulate that EC contractile force is being leveraged to locally restrict the size of the invasion pore, which if left unchecked, could cause irreparable damage to the EC.

**Figure 6:**
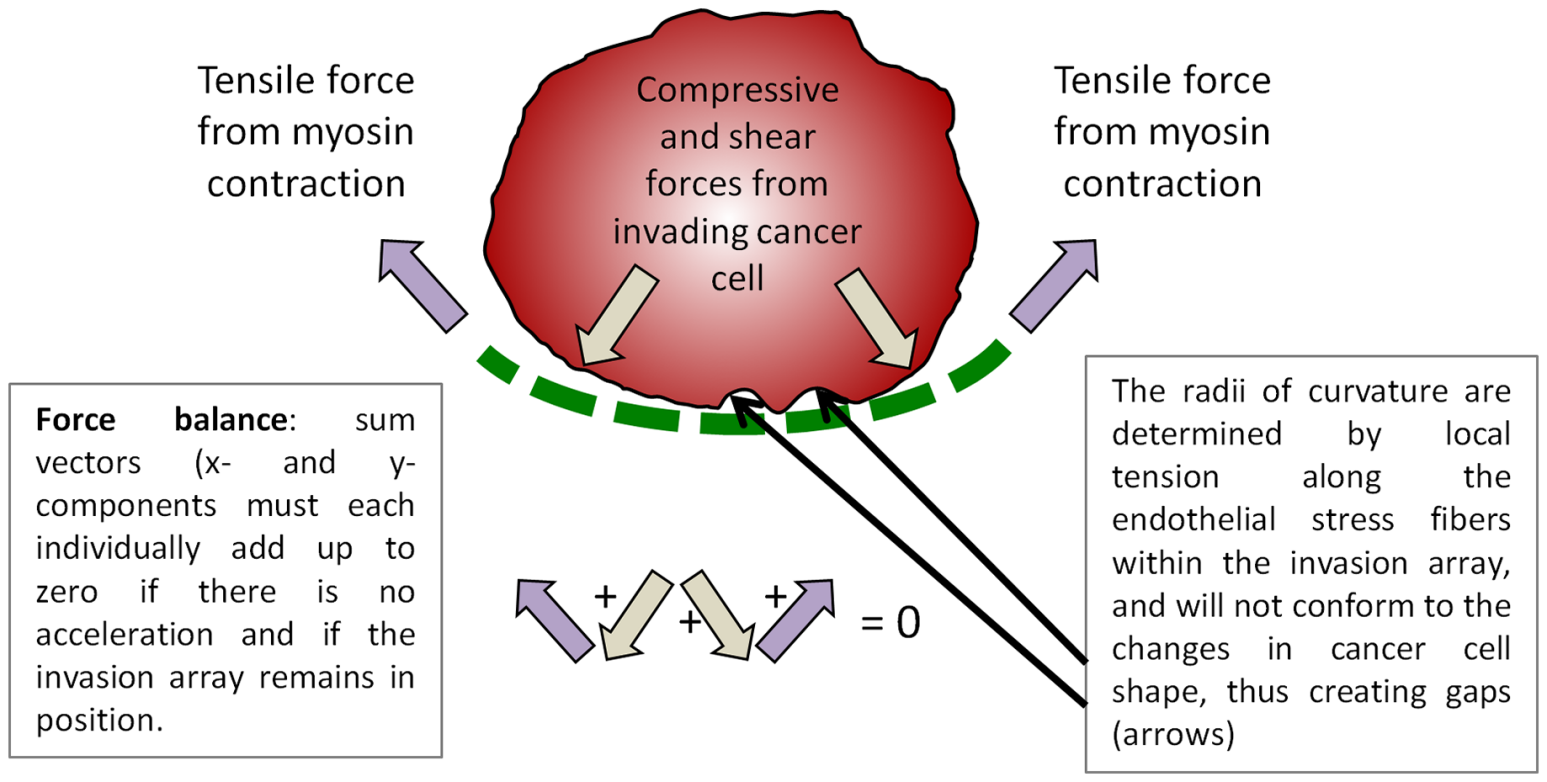
Schematics depicting the postulated balance of mechanical forces during invasion. At the onset of invasion the EC contractile force is overcome by the compressive force from the cancer cell, and the invasion pore grows in size, characterized by sarcomeric stretching and stress fibers breakage. As the EC recruits myosin for the TCIA assembly, the tensile force begins to counter the compressive force and the size of the invasion pore stays relative constant until the cancer cell exits the microwound, and the force balance reverses.

In this report, we also probed the relationship of viscoelasticity within a curved structure by laser-targeted photoablation of individual TCIA actomyosin fibers adjacent to the invading cancer cells. We revealed that the curvature of the TCIA was tension driven and that the EC cytoskeletal network would actively maintain the angle of the curvature. There is an apparent transference of tensile loads borne by the pre-ablated fibers to neighboring fibers upon scission. Stress fibers have been shown to possess the characteristics of the Kelvin-Voigt viscoelastic model [31]. The rapid relocation of the adjacent EC stress fiber to the exact position occupied by the previously severed fiber suggests a force balance that is dynamically maintained locally at the invasion site. In figure 6, we propose that the EC must counteract two biomechanical forces from the invading cell: (i) the compressive stress that is directed radially outward from the cancer cell as it seeks to enlarge the invasion pore, and (ii) the lateral shear stress as the cancer cell changes its shape and scrapes tangentially against the EC. Stress fibers are known to be sensitive mechanosensors whose abundance, structure and organization is modulated in part by biophysical signals between cells [32]. The diminished turnover rate within the TCIA thus strengthens the notion that the highly compressed and committed actomyosin system has been reorganized around the cancer cell. Ultimately, the increased mechanical stress imparted by the cancer cell is countered by the increased strain rate of the actomyosin fibers within the TCIA, which will generate an axial force that will locally push back against the cancer cell.

Strikingly, as shown by the many examples presented in this report and elsewhere [18], the edges of the transmigrating cell (cancer or leukocyte) and the underlying endothelial cell are often not packed tightly against one another. Instead, gaps can be clearly seen between the two cells within the invasion pore at the resolution of both electron microscopy and light microscopy. How the existence of gaps can convey biomechanical stress remains to be elucidated, but the fact that our measurements reveal a wide range of radii of curvature within individual TCIAs allows us to propose a potential explanation. It is likely that any given individual invasion network is made up of many actomyosin bundles akin to stress fibers [33], and that the arc angles are determined by the local balance of mechanical forces. We postulate that the arc angles are dictated by local Young's moduli of elasticity of the stress fibers. If our postulation is correct, then each segment can only bend to the optimally balanced angle even if some part of it is not touching the cancer cell.

Our data extends the notion of robust endothelial self-restorative capacity proposed by Martinelli *et al.* [10] in the late phase of diapedesis to include even earlier events. Therefore the protective remodeling response of the endothelial cell during transcellular diapedesis, at least in the case of tumor invasion, begins during the active transmigrating phase in the form of tension-driven cytoskeletal restructuring. Our observations highlight the fact that some of the endothelial responses to diapedesis are transient, localized and potentially too subtle to be accurately detected by conventional biochemical analyses. We acknowledge that data in this report do not include the potential roles of cytoskeletal components other than actin and myosin II. In fact recent reports from studies of leukocyte transcellular diapedesis suggest that microtubules, intermediate filaments and focal adhesions may also contribute to the TCIA [14], [34], [35]. Together, these observations strengthen several recent arguments [36]–[38] advocating the necessity of future paradigms to include biomechanics and physicochemical crosstalks as key elements to capture the complexity of transendothelial migration. Understanding endothelial cytoskeletal rearrangements during diapedesis expands our limited understanding of transendothelial invasion during cancer metastasis, but may also give insight into the role of the endothelium in leukocyte transcellular diapedesis and inflammation.

## Materials and Methods

### Cell culture

Primary human vascular endothelial cells (HUVEC) were purchased from Lonza (C2519A). Cells were cultured in EGM-2MV media (CC-3202; Lonza), only passages 2–5 were used. Breast cancer cell MDA-MB231, were cultured in Leibovitz media (21083027; Gibco) supplemented with 10% FBS, and 1% penicillin streptomycin solution. 293A cells were grown in DMEM (11965; Gibco) supplemented with 10% FBS, and 2% L-glutamine. All cells were cultured at 37° and 5% CO_2_, with the exception of MDA-MB231 cells which were cultured at 37° but with normal atmospheric levels of CO_2_ and O_2_. For immunofluorescence experiments ECs were plated on no. 1.5 coverslips (16004–302; VWR) or Matek dishes (P35G-1.5-14-C) coated with purified rat tail collagen.

### Plasmids

pAD-eGFP-RLC, was generated using the pAd-CMV-V5-DEST™ Gateway Vector Kit (V493-20; Invitrogen) according to manufacture's protocol from the original pEGFP-RLC plasmid as previously described [8].

### Adenoviral infections

Adenovirus was generated according to Invitrogen's Virapower Adenovirus Expression Protocol in 293A cells. To infect endothelial cells 0.5–10 ul of amplified virus was added to 1.5 ml of media in a 35 mm dish in media. After 24 hours the media was replaced and cells were maintained until they became confluent (usually 2 more additional days) at which point they were used for invasion assays.

### Cancer cell invasion assays

Cancer cells were labeled for 20 minutes with CellTrace Violet (1 ul per ml of PBS) (C34557; Invitrogen), rinsed twice with PBS and then allowed to sit 20 minutes in normal growth media. For timelapse live cell imaging experiments, cancer cells with labeled with PKH26 kit from Sigma according to manufacture's protocol. For imaging the gaps between the cancer cells and ECs, cancer cells were labeled with CellTrace Violet (2 ul per ml of PBS) and ECs were labeled with CSFE (3 ul per ml PBS) (C34554; Invitrogen) and a non-blocking Dylight 550 conjugated antibody to VE-Cadherin (a gift from Dr. William A. Muller, Northwestern University). Cells were trypsinized, washed with media, resuspended in complete EGM-2MV media, and added to the endothelial monolayer. Approximately 1.5–2.5 hours following the addition of the cancer cells, invasions were stopped by fixation (see below). For live cell imaging, cancer cells were trypsinized and added to a GFP-RLC expressing EC monolayer in EGM-2MV media and allowed to attach in a incubator CO_2_ at 37° for 30 minutes before being transferred to a Tokai Hit heated stage top CO_2_ incubator on the confocal microscope.

### Immunofluorescence

Cells were fixed in 3.7% formaldehyde for 5 minutes, permeabilized with 0.05% NP40 in TBS, and stained with VE-Cadherin and AlexaFluor568 (A11031; Invitrogen), for 30 minutes at 37°. Samples were mounted in Mowiol with 2.5% Dabco prior to imaging.

### Confocal imaging

Laser scanning confocal images were captured using Nikon Elements software on a Nikon A1R confocal microscope using a PlanApo λ100X 1.45 NA objective lens (Nikon). Parameters were set to satisfy the Nyquist criterion. Spinning disk confocal microscopy was performed on an Andor XDI Revolution (Andor Technology). The cells were imaged with a Nikon Plan Apo 100×1.49NA TIRF objective every 4 minutes. Images were acquired using MetaMorph with an Andor Neo cMOS camera.

### Fluorescence recovery after photobleaching

Live HUVEC monolayers expressing GFP-RLC and labeled with VE-Cad Dylight 550 were imaged using a Nikon A1R laser scanning confocal microscope with a PlanApo λ100X 1.45 NA objective lens (see above). At least two pre-bleach images of invasions or normal stress fibers were acquired. Photobleaching of GFP- RLC was performed for 5 iterations with 100% 488 nm laser transmission at 1.9 μs pixel dwell. We have conducted numerous prior experiments to confirm that our photobleaching condition will photobleach the targeted zone when observed with maximal pinhole size. Image acquisitions of fluorescence recovery were performed 15–20 seconds following initial FRAP for 5 min to obtain more data points within the linear range of the recovery curve to increase confidence of our k-value calculations. Imaging intervals were then increased to every 45 seconds to assure complete recovery following photobleaching. The intensity within the bleached region and a non-bleached stress fiber (as a control) were first measured using Fiji/ImageJ and then plotted with the FRAPCalc program (Dr. Rolf Sara, Åbo Akademi, Turku, Finland) to obtain the mobile fraction, t_1/2_, and k values. Statistical significance between TCIA and stress fibers was determined using an unpaired t-test.

### Laser ablation

Invasions were performed as described above on ECs grown on glass bottom Matek dishes. At approximately 90 minutes, cells were transferred to a Tokai Hit heated stage top CO_2_ incubator on the Nikon A1R microscope. An invasion event was located and an ROI was drawn over single stress fibers in each experiment. The ablation was performed with a 405 nm laser (100 mW Obis, Coherent Laser Group) through a Plan Apo 100×/1.45NA TIRF objective lens (Nikon) on a confocal laser scanning microscope (A1, Nikon). Full laser power for 2 seconds was used. The sample power (actual laser power exerted on the specimen) was estimated to be 2.1 mW. This value was derived from (i) actual laser power measurements with no lens present, (ii) actual lens-related transmission reduction measurements provided by Nikon USA, (iii) the size of the lens entrance aperture relative to the laser beam diameter, and (iv) laser beam diameter size derived from curve-fitted data of five Nikon objective lenses. We have avoided using the laser power measurement directly through the objective lens, since by doing so we would be measuring the laser transmission through air. This is because high N.A. lenses (above ∼0.95) will trap a fraction of the laser power due to total internal reflection, thus will under-represent the actual sample power when appropriate fluid immersion is used [39].

### Shape analysis

A region of interest was drawn lining the pore, the ROI was then analyzed in Fiji/ImageJ using the Shape Descriptor 1u plugin [40]. The trace of the pore was also fitted to a circle or ellipse using Fiji/ImageJ.

### SIM imaging and sarcomeric spacing analysis

For SIM imaging cells were imaged on a Nikon N-SIM system using a Nikon Plan Apo 100×1.49NA TIRF objective. Images were captured using Nikon Elements software with an Andor iXon3 camera. Z-step size was set to 0.100 um (well within Nyquist criterion). 15 images per slice (5 phases, 3 angles) were captured and then reconstructed using the Nikon Elements software. To minimize saturated pixels for quantification of the myosin spacing in different structures, we obtained images of the same field with various exposures. Images are displayed as extended depth focused maximum intensity projections. Sarcomeric distances were measured using intensity profiles drawn along the invasion pore. The intensity profiles passed through the center of each myosin head and spacing between peaks on the intensity profile indicated sarcomeric spacing. Statistical significance was determined by performing an ordinary one-way ANOVA in combination with Tukey's multiple comparisons in GraphPad Prism.

### Platinum replica

ECs were plated on no. 1.5 coverslips in 35 mm tissue culture dish and invaded with MDA-MB231 cells as described above. Coverslips were quickly rinsed with PBS to wash out any cells that were still floating in the growth medium. Cells were then extracted for 10 minutes at room temperature with 1% Triton x-100 in PHEM buffer (60 mM PIPES, 25 mM Hepes, 2 mM MgCl2, 10 mM EGTA, pH 6.9) which contained 5 mM phalloidin (Sigma). Extracted cells were quickly rinsed with PHEM buffer to remove extraction buffer and membrane debris. Samples were then fixed in a mixture of 4% formaldehyde and 2% glutaraldehyde in 0.1 M sodium cacodylate, pH 7.2 for 20 minutes at room temperature. Following fixation, samples were processed in the following order at room temperature: rinsed 3 times 5 minutes with TBS, stained for 20 minutes in 0.1% tannic acid in ddH_2_O, rinsed 3 times 5 minutes with ddH_2_O, stained for 20 minutes with 0.2% uranyl acetate in ddH_2_0, rinsed 3 times 5 min in ddH_2_O. Then samples were dehydrated in 10%, 20%, 40%, 60%, 80% and twice in 100% ethanol, for 5 minutes at each step. They were re-stained with 0.2% uranyl acetate in 100% ethanol for 20 minutes. Afterward, they were rinsed twice for 5 minutes in 100% ethanol and twice for 5 minutes in 100% ethanol that was kept in molecular sieve. Next samples were further dehydrated in a Tousimis Samdri-790 critical point dryer. Dehydrated samples were coated with platinum and then carbon using Bal-Tec Med 020 vacuum evaporator. Coated samples were floated on 30% hydrofluoric acid and then transferred to EM grids. Images were acquired using FEI Tecnai Spirit G2 120kV transmission electron microscope.

## Supporting Information

Figure S1 Paracellular and transcellular invasion of MDA-MB231 cells in HUVEC monolayers. Anti-VE-Cadherin (VE-Cad) immunofluorescence allowed us to distinguish paracellular invasion (with perturbed EC border) and transcellular invasion (with intact EC border). Confocal and orthogonal micrographs of paracellular (A–C) and transcellular invasion (D–F). (A–F) MDA-MB231 (Blue) ECs expressing GFP-RLC (green) stained with VE-Cad (red). Scale bar, 10 μM.
(TIF)

Movie S1 Spinning disk confocal video of paracellular invasion of MDA-MB231 into a HUVEC monolayer corresponding to Figure 1, D and E. MDA-MB231 (red) invading a HUVEC monolayer expressing GFP- RLC (green). Inverted image of GFP-RLC is shown alongside color image. Images were captured every 4 minutes.
(AVI)

Movie S2 Spinning disk confocal video of transcellular invasion of MDA-MB231 into a HUVEC monolayer corresponding to Figure 1, F and G. MDA-MB231 (red) invading a HUVEC monolayer expressing GFP- RLC (green). Inverted image of GFP-RLC is shown alongside color image. Images were captured every 4 minutes.
(AVI)

Movie S3 Serial laser ablations of curved stress fibers during invasion corresponding to Figure 5A. MDA- MB231 (red) invading a HUVEC monolayer expressing GFP-RLC (green). Arrowhead indicates ablation point. Time points for capture are indicated as time stamps on video as hh:mm:ss.
(AVI)

Movie S4 Single laser ablation of linear stress fibers during invasion corresponding to Figure 5B. MDA-MB231 (red) invading a HUVEC monolayer expressing GFP-RLC (green). Arrowhead indicates ablation point. Timepoints for capture are indicated as timestamps on video as hh:mm:ss.
(AVI)

Movie S5 Laser ablation of a single stress fiber from a resting HUVEC cell expressing GFP-RLC (green) corresponding to Figure 5C. Arrowhead indicates ablation point. Timepoints for capture are indicated as timestamps on video as hh:mm:ss.
(AVI)
