# Supplementary figures and images for: Structure and Biomechanics of the Endothelial Transcellular Circumferential Invasion Array in Tumor Invasion
Source: PLoS One. 2014 Feb 24;9(2):e89758. doi: 10.1371/journal.pone.0089758 (PMC3933692; doi:10.1371/journal.pone.0089758)

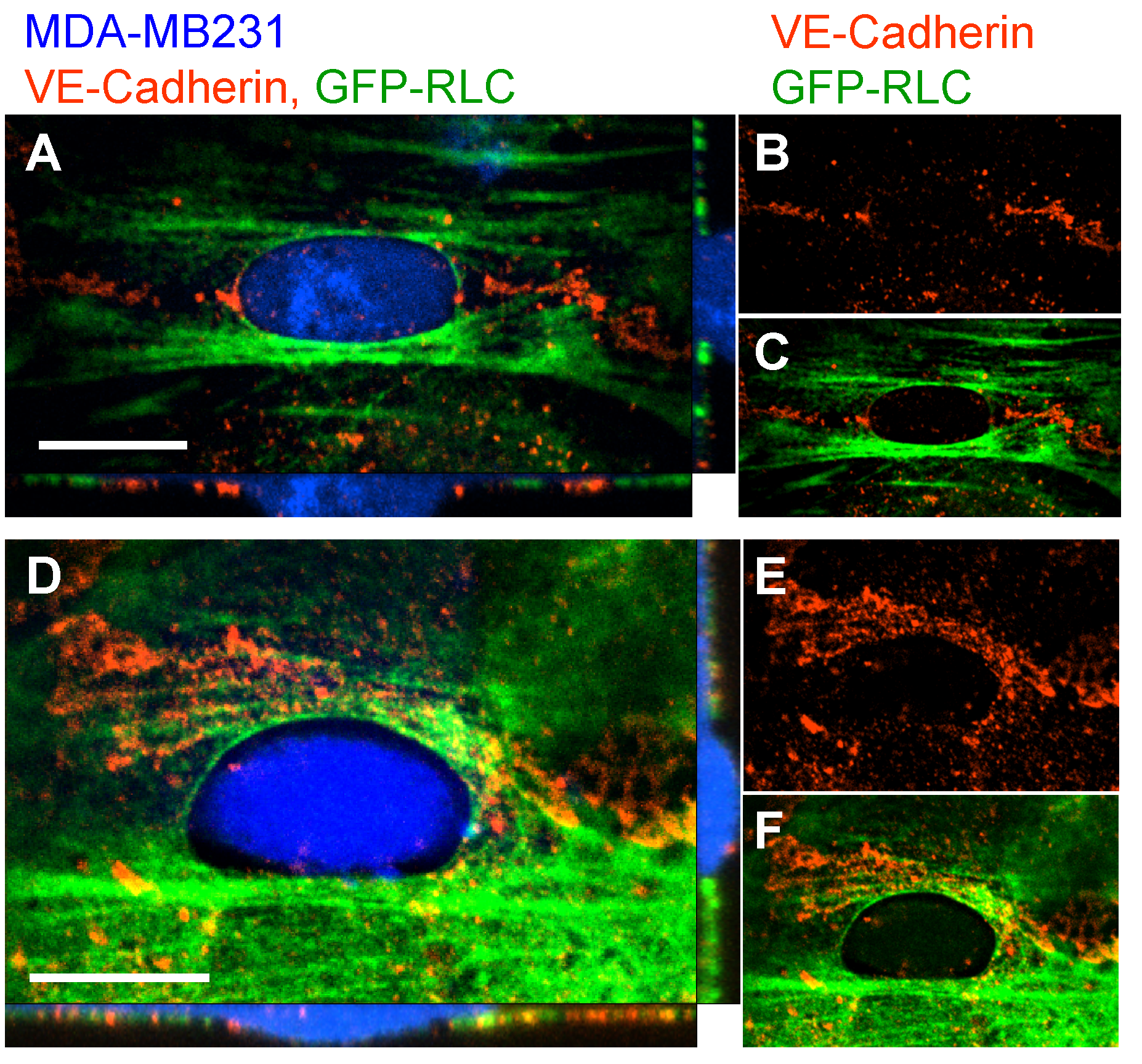

Supplement: Figure S1 — Paracellular and transcellular invasion of MDA-MB231 cells in HUVEC monolayers. Anti-VE-Cadherin (VE-Cad) immunofluorescence allowed us to distinguish paracellular invasion (with perturbed EC border) and transcellular invasion (with intact EC border). Confocal and orthogonal micrographs of paracellular (A–C) and transcellular invasion (D–F). (A–F) MDA-MB231 (Blue) ECs expressing GFP-RLC (green) stained with VE-Cad (red). Scale bar, 10 μM. (TIF) [file pone.0089758.s001.tif]
